# A Review of Cerebrospinal Fluid Circulation and the Pathogenesis of Congenital Hydrocephalus

**DOI:** 10.1007/s11064-024-04113-z

**Published:** 2024-02-10

**Authors:** Mingzhao Zhang, Xiangjun Hu, Lifeng Wang

**Affiliations:** Laboratory of pathology, Beijing Institute of Radiation Medicine, 27 Taiping Road, Beijing, 100850 China

**Keywords:** Cerebrospinal fluid circulation, Choroid plexus, Congenital hydrocephalus, Cilia, Aqueductal stenosis

## Abstract

The brain’s ventricles are filled with a colorless fluid known as cerebrospinal fluid (CSF). When there is an excessive accumulation of CSF in the ventricles, it can result in high intracranial pressure, ventricular enlargement, and compression of the surrounding brain tissue, leading to potential damage. This condition is referred to as hydrocephalus. Hydrocephalus is classified into two categories: congenital and acquired. Congenital hydrocephalus (CH) poses significant challenges for affected children and their families, particularly in resource-poor countries. Recognizing the psychological and economic impacts is crucial for developing interventions and support systems that can help alleviate the distress and burden faced by these families. As our understanding of CSF production and circulation improves, we are gaining clearer insights into the causes of CH. In this article, we will summarize the current knowledge regarding CSF circulation pathways and the underlying causes of CH. The main causes of CH include abnormalities in the FoxJ1 pathway of ventricular cilia, dysfunctions in the choroid plexus transporter Na^+^-K^+^-2Cl^-^ contransporter isoform 1, developmental abnormalities in the cerebral cortex, and structural abnormalities within the brain. Understanding the causes of CH is indeed crucial for advancing research and developing effective treatment strategies. In this review, we will summarize the findings from existing studies on the causes of CH and propose potential research directions to further our understanding of this condition.

## Introduction

The brain, as the central nervous system’s most advanced and structurally complex organ, is responsible for crucial functions such as sensation, learning, memory, and regulation [1]. Within the human brain, a group of cavity structures collectively known as the ventricular system exists, comprising the lateral, third, and fourth ventricles, interconnected through foramina. These ventricles are filled with a clear and transparent fluid called cerebrospinal fluid (CSF), which serves multiple roles including the removal of metabolic wastes from the brain, maintenance of environmental homeostasis, and regulation of intracranial pressure balance. The presence of CSF, a distinct fluid within the brain, has been recognized by humans for centuries. As early as the 2nd century A.D., Claudius Galen accurately described and documented the existence of a clear fluid inside the brain [2]. In a monumental contribution to the field, Leonardo da Vinci created the first illustration of the dissected ventricular system of the human brain in 1510, significantly advancing our understanding of ventricular anatomy [3]. In the 16th century, Swedenborg made noteworthy anatomical observations, shedding light on the brain, spinal cord, and blood circulation [4]. Magendie, a renowned anatomist of the 17th century, made significant advancements in the field of CSF research. His studies provided a deeper understanding of the anatomical structure of the brain, leading to the discovery of Magendie’s foramen, which serves as a connection between the fourth ventricle and the subarachnoid space. Furthermore, Magendie proposed the concept of CSF circulation within the ventricles, emphasizing its crucial role in maintaining brain homeostasis [5]. Building upon these findings, Faivre proposed the concept that CSF is produced by the choroid plexus (CP) [6]. In 1916, Haliltburton provided a comprehensive description of the major components of CSF, including proteins, glucose, and inorganic salts, in his book [7]. These historical milestones have played a crucial role in establishing our current understanding of CSF.

Hydrocephalus, a prevalent neurological condition, is characterized by the abnormal accumulation of CSF within the brain’s ventricles. This accumulation leads to ventricular dilatation, damage to the brain tissue, and the manifestation of various symptoms. Hydrocephalus can be classified clinically into two main categories: congenital hydrocephalus (CH) and acquired hydrocephalus, based on the time of onset. CH, a condition characterized by the presence of hydrocephalus symptoms in infants during prenatal development. In contrast, acquired hydrocephalus can occur at any given time following birth. Understanding the distinct characteristics and classifications of hydrocephalus is crucial for accurate diagnosis and appropriate treatment planning. In fact, the understanding and treatment of hydrocephalus by humans date back to ancient times. As early as the 2nd century common era (BCE), CH was described by Hippocrates, who is thought to have been the first to use ventriculostomy [4]. In the 10th century, Abulcasis recorded the first surgical method for treating infantile hydrocephalus, which involved opening the skull to drain the fluid from the brain—an undoubtedly bold attempt [8]. The first documented case of ventriculoperitoneal shunting, a procedure in which the ventricles are drained externally, was performed by Le Cat in 1744, marking him as the inventor of this technique. In the 19th century, West and Cheyne differentiated between acute and chronic hydrocephalus, recognizing both acquired and congenital causes of the condition [9]. Numerous treatment methods were explored in the quest to find a solution for hydrocephalus, encompassing the use of medications, diuretics, intracerebroventricular iodine injections, head wraps, bloodletting, and craniotomy. It was not until the groundbreaking year of 1891, when Quincke introduced lumbar puncture as an effective therapeutic approach for hydrocephalus, that clinical treatments began to center on the principle of CSF drainage as the primary intervention [10, 11]. In the same year, Walter invented the ventricular anastomosis, affirming in principle the validity of Quincke’s lumbar puncture [12]. The invention of the ventricular sulcus anastomosis was a major milestone in the exploration of treatment options for hydrocephalus. It opens up a shunt channel for excess CSF, which reduces intracranial pressure and alleviates the symptoms associated with hydrocephalus. Ventriculoatrial anastomosis has been improved and refined over time. Commonly used techniques for modern ventricular anastomoses include ventriculoperitoneal shunting and ventriculoatrial shunting. In 1941, Campbell introduced the closed, sterile External Ventricular Drainage System for the first time. This system allowed the drainage of CSF by inserting a drainage tube into the ventricle. Its introduction led to a significant decrease in infection rates related to conventional CSF drainage techniques and continues to be widely used in neurosurgical treatment today [13]. As early as the 1980s, ultrasound was able to assist doctors in diagnosing hydrocephalus in newborns [14]. In the present day, thanks to the advancement of imaging technology, hydrocephalus can be evaluated using CT and MRI scans, which offer detailed structural images. These imaging tools assist doctors in gaining a better understanding of the patient’s pathology, determining the severity and type of the disease, and consequently, making more precise treatment recommendations [15–17].

In the late 20th century, the clinical management of hydrocephalus involved the use of ventricular shunt, lumbar puncture with acetazolamide, furosemide, and other drugs, as well as diuretics [18–21]. With the relentless advancements in science and technology, endoscopic surgery has emerged as a widely adopted approach in the field of neurosurgical clinical treatment. This technique boasts several advantages, including minimal tissue damage, expedited recovery, and enhanced visibility. Endoscopic third ventriculostomy is a technique that effectively establishes a new passage within the ventricular system. This novel pathway allows cerebrospinal fluid to bypass any obstructed routes and directly drain from the ventricles. Endoscopic third ventriculostomy is primarily recommended for cases of obstructive hydrocephalus, where the underlying cause of the condition is clearly identified, and the anatomy of the cerebrospinal fluid circulatory system is suitable for the procedure [22, 23]. On the other hand, external ventricular drainage involves the insertion of a catheter into the ventricular system to facilitate the drainage of cerebrospinal fluid outside the body. This procedure is commonly employed for acute traffic hydrocephalus and serves as a temporary measure to alleviate pressure within the ventricles before definitive treatment is undertaken [24].

Currently, endoscopy is commonly employed in various procedures such as third ventriculostomy and ventricular shunting. Moreover, the field of contemporary medicine continues to investigate potential medications for aiding in the treatment of hydrocephalus. James et al. discovered that decorin possesses the ability to mitigate astrocyte hyperplasia resulting from hydrocephalus, thus offering potential therapeutic benefits for alleviating the pathological symptoms associated with this condition [25]. Furthermore, it can be effectively combined with choroidal plexus cautery to treat hydrocephalus [26–28].

While modern medicine has made significant strides in diagnosing hydrocephalus through advanced imaging techniques and providing relief through drainage and pharmacological interventions, the prevention and effective management of hydrocephalus still remain elusive. Hydrocephalus presents formidable medical challenges in developing countries due to high birth rates and limited access to medical resources. According to statistics, in Brazil, hydrocephalus causes the death of 1.5 out of every 100,000 newborns [29]. In Africa, the prevalence of CH can reach as high as 0.0145% [30, 31]. A recent study conducted in 2020 estimated that the total cost of hospital admission for a single hydrocephalus patient in a high-income country ranges between $10,000 and $14,000. In sub-Saharan Africa, the annual cost of treating hydrocephalus cases alone amounts to a staggering $190 million [32, 33]. This places an immense financial strain on families and patients, while simultaneously posing an enormous challenge to the healthcare systems of developing nations. The investigation into the etiology of CH is a pressing matter that can establish a fundamental scientific foundation and methodology for its prevention and treatment. In this review, we summarise existing theories on the production and circulation of CSF and the causes and molecular mechanisms of CH. Furthermore, we propose potential directions for exploring the causes of CH.

## Cerebrospinal Fluid

CSF plays a crucial role in the early development of the cerebrum, particularly in neurogenesis. CSF originates from neuroepithelial precursors, and during the early stages of brain development, it exerts pressure on the neuroepithelial wall within the ventricles. This positive pressure acts in coordination with the growth of neuroepithelial cells, contributing not only to morphogenesis but also to brain regionalization [34]. In addition to its role in promoting brain development, CSF creates a beneficial environment for the survival and growth of neural stem cells. It enhances the survival rate of neural stem cells, facilitates their differentiation, and promotes the generation of astrocytes. This supportive environment provided by CSF fosters the proper development and function of neural cells in the brain [35].

Gato et al. [36] explanted organ-cultured chicken embryonic mesencephalic neuroepithelial cell investigate the intrinsic capabilities of neuroepithelial cells in vitro. The results clearly demonstrated that neuroepithelial cells alone are insufficient to induce cell survival, replication, and neurogenesis. The researchers reached the important conclusion that the occurrence and proliferation of neuroepithelial cells are predominantly driven by extraneural signals present in the embryonic CSF (E-CSF) in the form of diffusion factors. Subsequent studies have consistently supported and validated this finding. It has been observed that fibroblast growth factor-2, which is present in the embryonic serum, is transmitted to the E-CSF through the neuroepithelium in vivo. This transmission of fibroblast growth factor-2 plays a direct role in the proliferation of epithelial cells and neural differentiation. Moreover, the neurogenic signals present in the E-CSF have been found to enhance the physiological activity of neural stem cells and induce the formation of neurons with normal migration patterns [37–39]. These collective findings underscore the critical role of extraneural signals in the E-CSF, in orchestrating the intricate processes of cell survival, replication, and neurogenesis during early brain development.

### Production of CSF

In an adult, the volume of CSF is approximately 150 ml, with a turnover rate of four times within 24 h, resulting in a production rate of 400–600 ml per day [40]. When the foetus is in the mother’s womb, CSF is secreted mainly through the embryonic soft meninges. After birth, CSF is secreted mainly by the CP. However, due to the incomplete maturation of both the CP cells and the ventricular system in newborns, the amount of CSF secreted is relatively small. In 1914, Cushing proposed the theory of CSF circulation, suggesting that CSF is secreted by the cells of the CP [41]. Subsequently, in 1919, Dandy demonstrated abnormal CSF circulation in a ventricle that contained a plexus after removing the CP on one side of the lateral ventricle and blocking the outflow channels [42]. This classic theory of CSF circulation gained wide acceptance. However, recent advancements in our understanding of glial lymphatic circulation have led to new discoveries regarding the origins of CSF. It is now known that CSF is not solely secreted by CP cells but also results from capillary ultrafiltration and exchange of interstitial fluid in brain tissues. This expanded understanding has broadened our knowledge of the complex processes involved in CSF production and circulation. Overall, these findings highlight the intricate nature of CSF dynamics and emphasize the importance of considering multiple mechanisms, including CP secretion and glial lymphatic circulation, in our understanding of CSF physiology.

#### CSF is Produced by the CP

The CP is a network of capillaries attached to the inner wall of the ventricles of the brain, which is covered by a layer of tightly connected, polarised epithelial cells [43]. The CP plays a crucial role in establishing a barrier between the ventricles and the blood. This barrier is supported by tight junctions in the CP epithelium cells and a complex network of ion channels and transporter proteins on the surface [44]. Through this mechanism, the CP ensures that ion concentrations within the CSF are maintained at optimal levels. Additionally, the presence of water channel proteins on the surface of epithelial cells facilitates the diffusion of fluids. The process of CSF formation primarily involves the net transport of Na^+^, Cl^−^, K^+^, and water from the plasma to the CP and subsequently into the CSF [45, 46]. Initially, ions and water are taken up at the basement membrane through facilitated mechanisms, then traverse the cytoplasm, and finally, are released or actively secreted into the ventricles. Furthermore, CP epithelial cells actively secrete specific molecules into the CSF. This process involves the transport and release of substances such as neurotransmitters, hormones and waste products, which play a crucial role in maintaining the overall function of the central nervous system and regulating long-range signaling within the brain [47–49]. CSF production is primarily attributed to the CP, as initially proposed by Cushing in the classical theory of CSF circulation [41]. The successful extraction of CSF from CP tissues of cats and rabbits, as well as the confirmed secretion of CSF by CP epithelial cells cultured in vitro, further support this notion [50–52].

#### CSF is Produced by Interstitial Fluid Transfer

However, with the development of medical research, it has been questioned that CSF is only produced in the CP. Bulat and Klarica proposed a new theory that CSF is produced not only in the choroid but also in the subarachnoid space. It states that the hydrostatic pressure between the capillaries in the subarachnoid space and the brain tissue space causes the capillaries to produce CSF by ultrafiltration [53].

In recent years, with the progress of technology, the phenomenon of liquid exchange between CSF and interstitial fluid (ISF) was found by fluorescence imaging and nuclear magnetic tracking technology, which proved that ISF was also one of the important sources of CSF. Jeffrey et al. first observed the exchange of fluid between subarachnoid CSF and ISF in living mice by two-photon laser scanning microscopy. They concluded that this exchange is mainly mediated by aquaporin-4 (AQP4) [54, 55]. AQP-4, which is abundantly expressed within the brain barrier, is believed to play a crucial role in regulating water dynamics specifically within the brain barrier. The astrocyte AQP-4 system actively facilitates the movement of interstitial fluid within the brain barrier. Its primary function is to ensure the proper flow of water into the pericapillary interstitial space, thus promoting the circulation of interstitial fluid [56]. Oernbo et al. found that the gradient of anti-penetration didn’t result in a serious impact on CSF secretion. Accordingly, they proposed a new theory that the secretion of CSF didn’t depend on traditional osmosis but was produced by the synergistic effect of the different choroidal transporters. Brain endothelial cells exhibit epithelial-like properties and display a polarized distribution of amino acid transporter proteins. The luminal membrane of these cells contains the Na/K-ATPase protein. This polarized distribution of ion transporter proteins may serve as the foundation for active fluid secretion across the blood-brain barrier [57]. Besides, they confirmed that Na^+^-K^+^-2Cl^−^ contransporter isoform 1 (NKCC1), Sodium bicarbonate cotransport and Na^+^/K^+^-ATPase may be key factors of CSF secretion [58].

### Circulation of CSF

Within the cerebral hemispheres, there exist a pair of symmetrical C-shaped cavities known as the lateral ventricles. These ventricles are connected to the third ventricle, which is nestled between the two thalami, through the mesencephalic foramina. In the midbrain, there exists a narrow canal called the midbrain aqueduct, which serves as a passage connecting the third and fourth ventricles. Ultimately, the fourth ventricle is linked to the subarachnoid space through the central foramen and two lateral foramina.

#### Classical Circulation of CSF

**Fig. 1:**
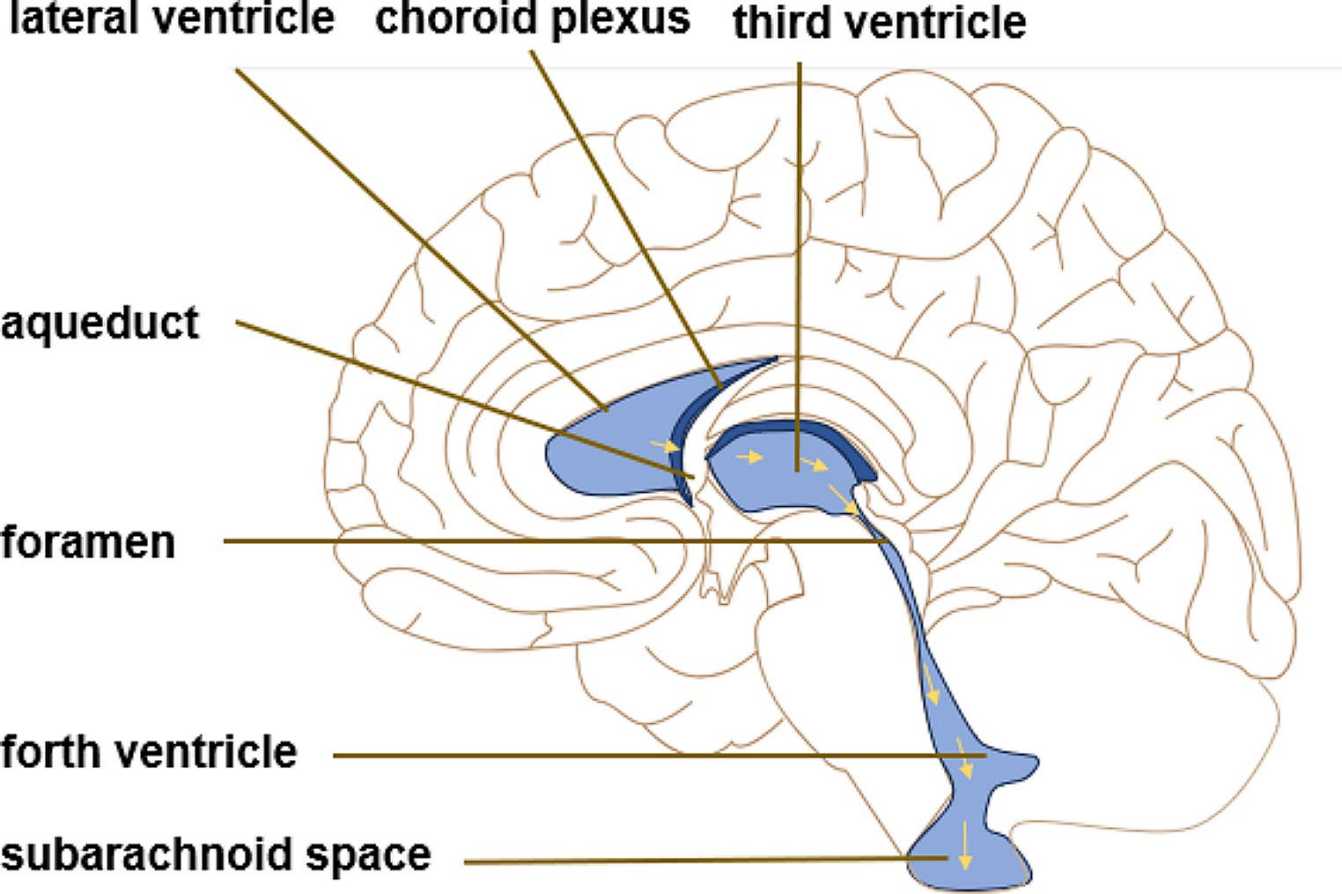
Classical CSF circulation theory. CSF is absorbed from the third ventricle and the fourth ventricle to the subarachnoid space through the CP secretion pathway. The yellow arrow is the direction of flow of CSF

The left and right lateral ventricles were distributed in the cerebral hemisphere and connected with the third ventricle through the interventricular foramen. The fourth ventricle is located between the cerebellum, medulla oblongata and brainstem, connected to the third ventricle through the aqueduct. Classical CSF flow hypothesis claims that CSF is pulsatile, which corresponds to the systolic pulse wave of choroidal artery [41]. CP cells orchestrate the production of CSF through their selective transport of ions and molecules, a process essential for maintaining the delicate balance of the central nervous system. Once created, CSF starting its voyage from the lateral ventricles and traverses the mesencephalic aqueduct, making its way to the third ventricle. After crossing the fourth ventricle CSF flows out of the ventricular system through the midbrain aqueduct and the Lucas’ foramen of the mesencephalon. Finally, part of the CSF is absorbed by the arachnoid granules and the rest flows into the central canal of the spinal cord, completing the process of a circulation of CSF [41, 59].(shown in Fig. 1).

#### Glymphatic Hypothesis

As glymphatic hypothesis proposed, glymphatic pathway provides a new direction for the exploration of CSF circulation mechanism. It states that CSF and ISF are exchanging and merging around the peripheral space of capillaries. Therefore, the circulation of CSF is likely to be based on the circulation of the whole flow under the power of glial lymphatic circulation. Although the theory has not been verified due to technical limitations, it has been found that the circulation of CSF and ISF is not only through molecular diffusion, but also a complex movement caused by pressure gradient, rotation repetition, acceleration and deceleration. In addition, CSF flow reverses around the subarachnoid artery and in the cortex. It means that the circulation of CSF is not just a simple circulation flow but combinations in ventricles and subarachnoid space in various directions [60, 61]. Salehi et al. [62] found that the number of cell deaths in embryos lacking CSF increased, so they indicated that normal circulation of CSF was very necessary for survival of developing cerebral cortical cells.

CSF is usually absorbed through arachnoid granulations by the internal jugular vein system. There are three types of arachnoid granules in the spinal cord: the first type is located inside the nerve root of cerebral dura meter, the second type reaches the cerebral dura mater, and the third type expands to the epithelial space through the cerebral dura mater. Standing posture under the action of gravity improve the absorption of CSF by the arachnoid villi in human lumbosacral nerve roots, then absorbed CSF enters the lymphatic system [63].

By employing computer simulation imaging techniques, Dacersin-Catty et al. discovered that the exchange of cerebrospinal fluid and cerebral tissue fluid within the Virchow-Robin space is governed by a combination of oscillatory and convective flow. This flow is regulated by the dilation of cerebral arteriolar walls, rigid movement, and static cerebrospinal fluid pressure gradients [64].

### Absorption of CSF

The arachnoid membrane is comprised of arachnoid cells that possess specialized microvascular structures called arachnoid villi, resembling small protrusions. These microvessels contribute to the formation of a blood-cerebrospinal fluid barrier through a layered arrangement of vascular endothelial and ectothelial cells.

Within the subarachnoid space, in addition to the arachnoid membrane, there are capillaries and astrocytes. Capillaries contain endothelial cells that express the water channel protein AQP-1 on their cell membranes, enabling the absorption of water from the cerebrospinal fluid. Astrocytes are connected to the endothelial cells in the subarachnoid space by peduncles, which serve as conduits for water molecules to enter the subarachnoid space. Subsequently, these water molecules are ultimately absorbed into the bloodstream [54, 65, 66].

In summary, the absorption of cerebrospinal fluid within the subarachnoid space involves the participation of capillaries, astrocytes, and the arachnoid membrane. These structures work in concert to ensure the circulation of cerebrospinal fluid and maintain fluid homeostasis within the brain.

## Mechanism Classification of CH

CH typically refers to hydrocephalus symptoms that arise from genetic inheritance, protein expression disorders, ventricular structural abnormalities, CSF circulation disorders, and regulatory dysfunction. It is commonly present at birth in infants. The excessive accumulation of CSF exerts pressure on the ventricles, resulting in their expansion and subsequent enlargement of the developing skull. This, in turn, causes traction and compression damage to the surrounding brain tissue.

Temporal changes in CSF dynamics and brain morphometric parameters have been observed in patients with hydrocephalus [67]. Seifollah et al. conducted a study utilizing imaging techniques to measure intracranial compliance, cerebrospinal fluid volume, and intracranial pressure in hydrocephalus patients who underwent a successful shunt procedure. Interestingly, they found that the restoration of intracerebral homeostasis was not immediate following shunt placement but instead took approximately 6 months to reach a stable state [68]. Furthermore, another study by Liu et al. employed Pearson’s correlation coefficient to establish a relationship between mean cerebrospinal fluid flow in the midbrain aqueduct and intracranial pressure. They discovered that patients with hydrocephalus exhibited specific cerebrospinal fluid dynamic parameters in the midbrain aqueduct, including peak positive velocity (7.348 cm/s), mean velocity (0.623 cm/s), and mean flow (50.799 mm3/s) [23]. These findings shed light on the intricate dynamics of cerebrospinal fluid in hydrocephalus patient.

There are two main categories of the hydrocephalus: obstructive hydrocephalus and communicating hydrocephalus. Obstructive hydrocephalus occurs the obstruction of the ventricular system or subarachnoid outlet, resulting in an excessive buildup of CSF. In communicating hydrocephalus, although there is an intact communication pathway between the ventricular system and the subarachnoid space, CSF is often overproduced or poorly absorbed, leading to insufficient venous drainage. CH can be caused by various factors, including congenital malformations, viral infections, inflammation, bleeding, tumors, metabolic abnormalities, and genetics [69, 70].

**Fig. 2:**
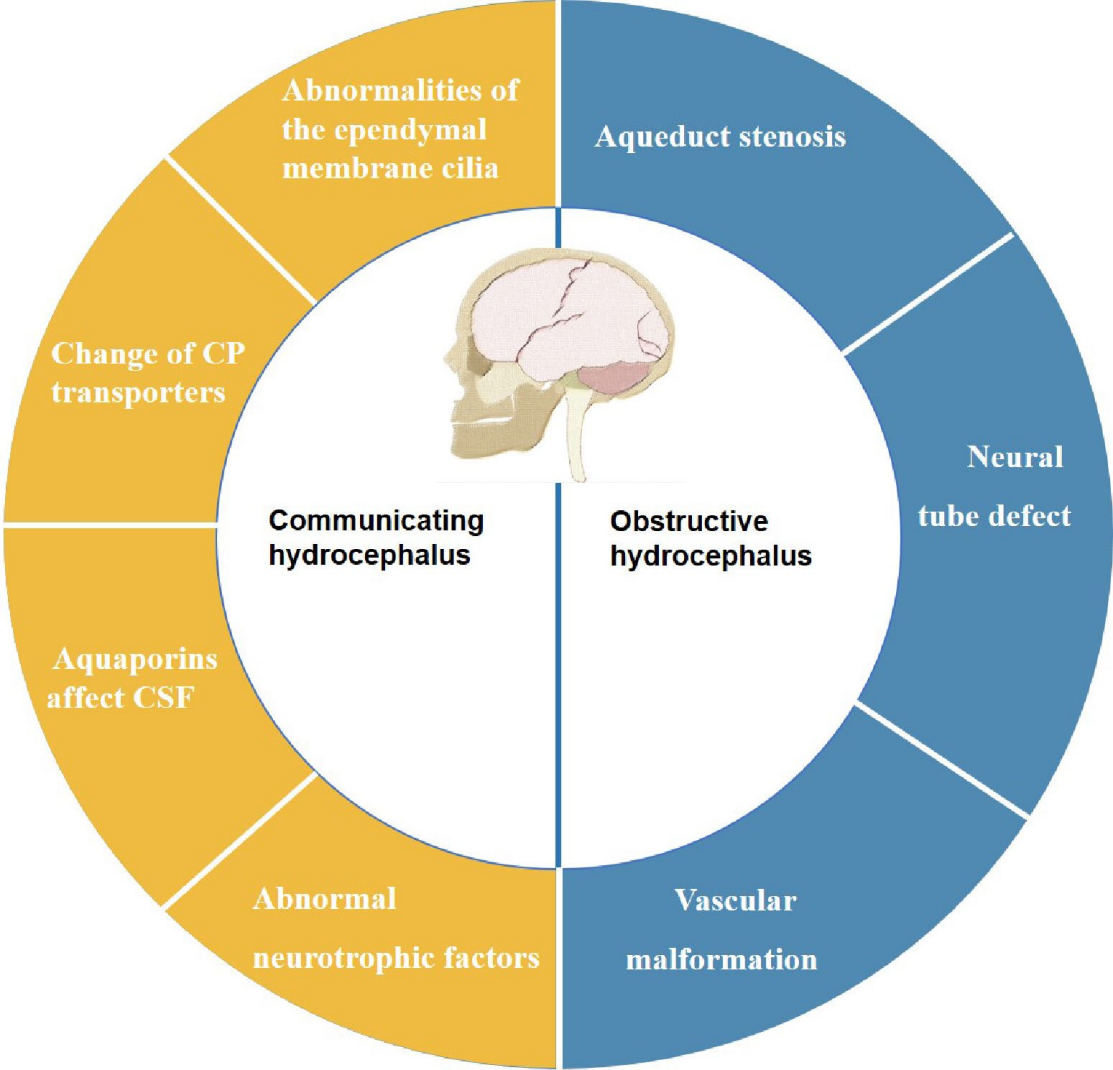
Causes of CH

Congenital obstructive hydrocephalus can have multiple causes, such as vascular malformations or venous dysfunction, ventricular or subarachnoid obstructions, and abnormal development of ventricular structures like congenital aqueductal stenosis, neural tube malformations, or congenital atresia of the Monro foramen [71] On the other hand, communicating hydrocephalus often arises from compromised CSF circulation due to insufficient power for CSF flow, which can be related to ependymal cilia movement, protein expression, and signaling pathways that regulate this process. Figure 2 illustrates these causes.

### Communicating Hydrocephalus

#### Abnormalities of the Ependymal Membrane Cilia Lead to Hydrocephalus Caused by Obstruction of CSF Flow

Cilia located on the surface of ventricular membrane cells are hair-like projections. Within the ventricles, ependymal membrane cells possess cilia that exhibit motor function. These cilia, powered by axial filament motor proteins and regulatory compounds, undergo reciprocal motion, effectively promoting the circulation of CSF. Yuzo et al. observed movement patterns of latex beads injected into CH HTX rats and WIC-HYD rats. The results indicated that disturbances in ependymal cilia movement are a significant contributing factor to hydrocephalus [72]. Ibanez et al. were the first to propose the concept of ependymal flow, particularly the directional flow of CSF generated by the movement of ependymal cilia [73].

Subsequent experiments have confirmed that inhibition of FoxJ1 protein expression leads to ventricular cilia deficiency, resulting in hydrocephalus. The p53 protein family is a sequence-specific transcription factor involved in the regulation of cell cycle, DNA repair, apoptosis and differentiation. Among them, p73 is a member of the p53 protein family and plays an important role in neural development. The p73 deficient mice showed hydrocephalus and cortical dysplasia. Marshall et al. analyzed p73-deficient mice and found that the number of multiciliated cells in mice decreased, which confirmed that p73 is a direct regulator of FoxJ1 and participates in multiciliated cells differentiation [74]. Medina et al. found ependymal denudation and cilia loss in p73 deficient mice. Although p73 is not an essential protein for ependymal cell differentiation and cilia formation, p73 determines the survival and long-term maintenance of the ependymal layer. Ependymal loss and CP disorder occurred in p73 deficient mice, which disrupted the dynamics of CSF flow and caused hydrocephalus [75].

In addition cilia with abnormal morphology or loss of motor ability can also cause hydrocephalus. The axial motor protein is an essential component of cilia and plays a crucial role in their motor function. Deletion of the Mdnah5 gene leads to specific abnormalities in the ultrastructure of ependymal cilia, suggesting that the Mdnah5 gene is vital for the structural integrity and proper functioning of these cilia. In Mdnah5 mutants, the cilia on ependymal cells can develop normally but are unable to move, resulting in impaired CSF flow and subsequent hydrocephalus [73].

In addition to the classical FoxJ1 pathway, there are other pathways that can act directly on cilia function. Planar cell polarity (PCP) and intercellular junction complex establish tissue structure and transepithelial coordination behavior. In multiciliary ependymal cells, rotated and translated PCP coordinate cilia swing and CSF circulation directly. PCP disruption leads to cilia lesions and hydrocephalus. There are two subtypes of p73, TAp73 with apoptotic activity and Np73 with anti-apoptotic function. TAp73 is glycosylated and secreted into CSF, which induces apoptosis through P53 target gene. Sandra et al. proved that TAp73 is an important regulator of PCP and regulates the occurrence of cilia [76]. In addition, Kif3a, Tg737, RSPH9, MT1-MMP, Cfap43, which are essential proteins for the formation of motor cilia, will lead to abnormal number or morphology of cilia and lead to hydrocephalus [77–84].

While several studies have suggested a correlation between abnormal ependymal cilia morphology and hydrocephalus, there remains considerable controversy regarding whether CH is directly caused by such abnormalities. It is important to note that ependymal cilia develop and mature gradually after birth, indicating that they likely do not play a significant role during the embryonic period [85]. In a recent publication by Duy et al. discussed their findings from studying multiple mouse models of hydrocephalus with mutated cilia genes. Interestingly, these animals exhibited hydrocephalus symptoms before the ependymal cilia had fully matured [86]. This suggests that abnormal cilia morphology and function may not be the sole cause of CH but rather could be one of the comprehensive effects resulting from hydrocephalus. As a result, further research is necessary to fully elucidate the role of abnormal ependymal cilia in the development of hydrocephalus.

#### Changes in Surface Transporters from CP

The transporters of CP facilitate two-way transport at the interface between the CP and CSF, driven by ion gradients. Maintaining a balance between CSF production and clearance.

NKCC1, expressed in the CP, plays a significant role in the formation of CSF by facilitating the transmembrane transport of Na^+^, K^+^, Cl^−^, and water. This enables water transport to occur independently of osmotic gradients, and in some cases, can even reverse them. During the early postnatal period, NKCC1 in the CP primarily regulates the clearance of CSF. Enhancing its function has been shown to alleviate ventriculomegaly symptoms in obstructive hydrocephalus models [87–89].

However, some research studies have indicated that the enhanced function of NKCC1 can, in certain cases, result in hydrocephalus symptoms. Transient receptor potential channel subfamily V member 4, an ion channel widely expressed in various tissues, is activated by chemical, osmotic, and mechanical stimuli. It plays a role in numerous pathological and physiological processes related to cell signal transduction by regulating calcium ions. Activation of transient receptor potential channel subfamily V member 4 by lipids can lead to the overactivation of NKCC1, resulting in excessive secretion of CSF and the development of hydrocephalus [90, 91].

Furthermore, the phosphorylation of NKCC1 can enhance ion coupling, which promotes transmembrane water transport in the epithelial cells of the CP, ultimately contributing to the pathogenesis of hydrocephalus. Non-receptor protein tyrosine phosphatases (PTPNs), involved in peptide-tyrosine dephosphorylation, regulate protein tyrosine phosphatase activity. Deletion of the ptpn20 gene in the PTPNs family leads to enhanced phosphorylation of NKCC1 cotransporters, thereby increasing CSF secretion and resulting in hydrocephalus [92, 93].

In summary, the regulation of CSF secretion and clearance by NKCC1 is a bidirectional process that can have varying effects on the symptoms of hydrocephalus, depending on the specific developmental stage and the main functions being performed. Understanding the intricate relationship between NKCC1 function and the development of CSF secretion and clearance is crucial and requires further exploration.

#### Aquaporins Affect CSF Secretion

Water homeostasis in the brain plays a crucial role in inducing and regulating water movement, which is achieved by controlling the expression and distribution of aquaporins. Aquaporins are proteins with aqueous pore structures found in cell membranes and barriers. In the human body, water channel proteins are widely distributed, especially in tissues specialised for secretion or fluid absorption [94].Among the aquaporins involved, AQP1 and AQP5 are key players in controlling water flux and CSF production within the CP barrier. Immunolabeling of AQP4 reveals its distinct polarization in perivascular astrocytes. On the other hand, AQP9 immunolabeling is primarily observed in the periventricular region of the parenchyma and the astrocytic protrusions at the edge of the subarachnoid space. This localization pattern suggests that both AQP9 and AQP4 contribute to the movement of water between the CSF and brain parenchyma [95].

Andrea et al. conducted a study that demonstrated elevated levels of AQP4 in patients with neurodegenerative diseases compared to those in healthy individuals [96]. This finding confirms the significant role of AQP4 in the exchange of body fluids within the central nervous system. Taehyung et al. induced acute hydrocephalus in rats by kaolin and the researchers observed a significant decrease in the expression of AQP1 in the CP during the early stages of the disease. However, in the later stages, there was an increase in AQP1 expression. Interestingly, the expression of AQP4 in the brain parenchyma did not show significant changes in the early stages of hydrocephalus but was found to be higher than in the control group during the late stages [97]. Furthermore, Shen et al. observed an increase in AQP4 expression in the brains of HTX rats with CH. This suggests that aquaporins may have a compensatory effect in the water physiology of hydrocephalus, indicating their potential role in regulating fluid balance in such conditions [98]. Taken together, these studies highlight the dynamic changes in the expression of AQP1 and AQP4 during the different stages of hydrocephalus. The findings also suggest a compensatory mechanism involving aquaporins in response to altered water physiology. Further research is needed to fully understand the precise role of aquaporins in the pathophysiology of hydrocephalus and their potential as therapeutic targets.

#### Abnormal Neurotrophic Factors

CSF contains various growth factors and neurotrophic factors that play crucial roles in promoting the survival and proliferation of nerve cells. Deprivation of nerve growth factor has been shown to induce apoptosis in developing cerebral cortex cells, emphasizing the importance of these factors. The neurotrophic protein family includes important members such as brain-derived neurotrophic factor (BDNF), nerve growth factor (NGF), neurotrophin-3, and neurotrophin-4 (NT-4).

BDNF, in particular, exerts its effects through the tropomycin receptor kinase B and the low-affinity p75 pan-neurotrophin receptor. It regulates neuronal differentiation and influences neurotransmission, specifically serotonin and dopaminergic neurotransmission. BDNF is a key mediator of functional and structural plasticity within the central nervous system [99]. In patients with normal pressure hydrocephalus, a decrease in BDNF levels has been detected in the CSF [100]. This suggests that insufficient neurotrophic support may be related to the development of hydrocephalus symptoms. Salehi et al. discovered that the lack of neurotrophic factors led to circulation disorders within the E-CSF, resulting in a decrease in cortical thickness and an increase in cell death. This phenomenon helps explain why the cortex of spina bifida fetuses is thinner, as the CSF fails to properly circulate throughout the ventricular area [62]. These findings highlight the critical importance of normal CSF circulation for the survival and development of cortical cells.

Masaki et al. conducted a study in which they investigated the expression levels of neurotrophic factor genes in the brains of rats with kaolin-induced hydrocephalus. They found that the expression level of NGF increased with the severity of hydrocephalus symptoms. Additionally, the expression levels of BDNF and its receptor, tropomycin receptor kinase B, increased over time [101]. These findings suggest that neurotrophic factors and their receptors are overexpressed in the damaged structures associated with hydrocephalus.

In another study by Frederike et al. it was observed that the levels of NGF and neurotrophin-3 in the CSF of children with hydrocephalus were significantly elevated. This indicates that a decrease in the expression levels of neurotrophic factors may lead to insufficient neurotrophic support in cases of CH, potentially contributing to the development of hydrocephalus. However, in acquired hydrocephalus, characterized by impaired CSF flow, compensatory upregulation of neurotrophic factor expression is observed [102]. These findings shed light on the role of neurotrophic factors in hydrocephalus and highlight the need for further research to fully understand their contribution to the pathophysiology of this condition.

### Obstructive Hydrocephalus

Obstructive hydrocephalus occurs when there is a blockage in the circulation pathway of CSF above the fourth ventricle, preventing it from flowing into the subarachnoid space as it should. This obstruction leads to an excessive accumulation of CSF, resulting in ventricular enlargement, increased intracranial pressure, and compression of the brain parenchyma. Common causes of obstructive hydrocephalus include abnormal ventricular structures such as aqueductal stenosis, obstruction of the Monro hole, neural tube malformations, and scar tissue blockage of the CSF circulation pathway caused by tumors, infections, inflammations, and other factors. Brain tumors have been found to be the main cause of hydrocephalus in children, accounting for as much as 50% of cases [103].

#### Aqueduct Stenosis

Aqueduct stenosis occurs when there is a narrowing or blockage of the cerebral aqueduct, which is a narrow channel connecting the third and fourth ventricles in the brain. This narrowing can be caused by various factors, such as developmental abnormalities, tumors, infections, or hemorrhages. The morphology of the aqueduct is determined by the tissue surrounding it. In a normal structure, the bottom of the aqueduct is triangular and lined with columnar cells. However, in cases of aqueduct stenosis, the ependymal cells surrounding the aqueduct undergo changes in morphology. Studies conducted on animal models, such as rats, have shown that aqueduct stenosis can lead to obstructive hydrocephalus. In these models, the ependyma surrounding the aqueduct undergoes remodeling, which results in changes to the shape and size of the aqueduct. It is believed that these changes are compensatory mechanisms aimed at maintaining CSF flow despite the narrowing of the aqueduct [104].

There are tiny organs formed by specialized ependymal cells on the walls of the third and fourth ventricles, which are called circumventricular organs (CVO). These CVOs are characterized by their abundance of neurons and glial cells and play important roles in neuroendocrine function and neuroimmunomodulation. Situated at key locations along the CSF circulation pathway, CVOs can be broadly classified into two categories: secretory CVOs, which release peptides, and sensory CVOs, which regulate signal transmission [105–107]. Among them, the subcommissural organ (SCO) is a highly conserved secretory CVO, which secretes a stromal cell protein SCO-spondin that can bind, regulate and transport different CSF molecules into CSF. The common cause of HTX hydrocephalus rat model is the atresia of aqueduct caused by SCO malformation, which leads to obstructive hydrocephalus [108, 109]. SCO-spondin can form a dynamic linear structure in the CSF known as the Reissner fiber (RF). The absence of RF leads to the malformation of embryonic axis development and aqueduct stenosis [110–112]. In hydrocephalus rats, a reduction in RF and p73 protein levels, as well as decreased SCO secretion, has been observed in the CSF. Additionally, the presence of antibodies against p73 protein and RF has been detected in both SCO and ependymal cells [113, 114]. Overall, malformations in the SCO and disruptions in SCO-spondin secretion have been closely associated with the development of aqueductal stenosis-induced hydrocephalus. Therefore, further investigations into the functions of CVOs and the molecules they produce hold great promise in deepening our understanding of the underlying pathophysiology of hydrocephalus and identifying potential therapeutic targets.

Basilar artery dilatation, venous malformation near the aqueduct, and the formation of the aqueduct network have been identified as factors contributing to poor CSF flow and the development of obstructive hydrocephalus [115–117].To address the challenges posed by aqueduct stenosis-induced hydrocephalus, clinical interventions such as third ventriculostomy and CSF shunt procedures are commonly performed. Third ventriculostomy involves creating an alternative passage for CSF drainage by surgically creating a small hole in the floor of the third ventricle. This allows CSF to bypass the obstructed aqueduct, reducing pressure and promoting improved fluid flow. CSF shunt procedures, on the other hand, involve the placement of a drainage system that diverts excess CSF to another body cavity, such as the abdominal cavity or the heart. This helps to alleviate the buildup of CSF in the brain, relieving hydrocephalus-related symptoms and reducing the risk of further complications [118–120].

#### Neural Tube Defect

Neural tube defect is a serious congenital defect of the central nervous system, which can lead to abnormal embryonic development, including spina bifida, anencephaly, obstructive hydrocephalus, ventriculomegaly, kidney malformation and cortical dysplasia [121–123].

The causes of neural tube defect are complex and diverse, including genetic factors, folic acid deficiency, and maternal diabetes. Extensive research has demonstrated the crucial role of folic acid in facilitating methyl transport during amino acid synthesis. Supplementation with folic acid during pregnancy has been shown to reduce the occurrence of fetal neural tube defects by approximately 70%. Furthermore, alterations in the glucose metabolism pathway have been implicated in the development of neural tube defects. Administration of multivitamins can help prevent embryonic malformations associated with diabetes [124, 125]. Key genes involved in the core planar cell polarity pathway and cell adhesion molecules, which mediate intercellular interactions, are believed to be important in neural tube formation [126, 127]. Understanding the intricate mechanisms involving these factors is essential for unraveling the complex processes underlying neural tube development.

#### Vascular Malformation

The human brain is an incredibly intricate and adaptable organ, playing a vital role in the functioning of the entire body. Proper nutrition delivery and waste metabolism in the brain rely on efficient blood transmission. Consequently, vascular malformation can significantly impact the normal physiological functioning of the brain. Developmental venous malformations, for example, can lead to aqueductal stenosis and subsequent obstructive hydrocephalus [128]. Vein of Galen aneurysmal malformations are a common type of congenital brain vascular malformation that often results in venous hypertension and defects in CSF absorption. This can lead to communicating hydrocephalus as the mass effect obstructs the normal circulation pathway. Currently, effective treatments for vein of Galen aneurysmal malformations include endovascular closure, CSF shunt procedures, third ventriculostomy, and endoscopic third ventricle-cisternal anastomosis [129–131].

Another well-known congenital malformation is Chiari malformation, with type I being the most frequently observed variant. Chiari malformation type I is characterized by the downward herniation of the cerebellar tonsils and vermis into the spinal canal, which can lead to obstructive hydrocephalus [132]. These conditions emphasize the critical importance of understanding and effectively managing congenital brain malformations. Advancements in treatment options, such as endovascular techniques and surgical procedures, have improved patient outcomes and quality of life. Continued research and clinical efforts in this field are vital for enhancing our understanding of these complex malformations and developing innovative therapeutic approaches.

## Concluding Remarks

As we have described in this review, the current widely recognised mechanisms and circulation patterns of CSF production remain the classical theories of Chusing. Besides, it is important to note that new discoveries, such as the interstitial fluid exchange mechanism and the glial lymphatic circulation hypothesis, require further validation through rigorous experimental studies. Throughout the history of CSF research, its exploration has often been closely linked to the study of hydrocephalus. By gaining a deeper understanding of CSF production and circulation, we can enhance our investigation into the causes of hydrocephalus, which is pivotal for the prevention and treatment of CH. Future research should focus on unraveling the complexities of CSF dynamics and its interactions with the brain’s interstitial fluid and lymphatic systems. By elucidating these intricate mechanisms, we can advance our knowledge of hydrocephalus pathogenesis and identify novel therapeutic approaches, ultimately improve patient outcomes and quality of life for individuals affected by CH.

In cases of CH, communicating hydrocephalus is primarily caused by abnormalities in cilia movement, alterations in the characteristics of CSF ion transporters, imbalances of aquaporins, and bnormalities in neurotrophic factors. These factors are closely linked to the period of brain and nerve development, and changes in gene expression during different periods can yield diverse outcomes. Therefore, a comprehensive understanding of the molecular mechanisms underlying CH necessitates a study that combines the investigation of these factors with the brain development process. During our literature review, we discovered that the down-regulation of FoxJ1 signaling is frequently implicated in the abnormal formation of cilia in ventricular cells. Investigating whether all perturbations in the upstream signaling of FoxJ1 are associated with hydrocephalus represents a promising avenue for future exploration. Furthermore, a recent study highlighted that abnormalities in CP cilia can also contribute to the onset of hydrocephalus, this may mean a new direction for research.

Furthermore, it is important to acknowledge the significant contribution of choroidal surface transporters, water channel proteins, and ion channels in the process of CSF secretion. These functional proteins are essential for maintaining the appropriate composition of CSF and regulating the volume of fluid secreted. Any abnormalities in these proteins can disrupt the delicate balance, leading to alterations in CSF composition and secretion. Exploring the development of therapeutic agents that target these functional proteins represents an emerging and promising area of interest for future hydrocephalus treatments. By identifying and modulating these key players, we may be able to restore normal CSF dynamics and improve outcomes for individuals affected by hydrocephalus.

Obstructive hydrocephalus, on the other hand, is primarily caused by changes in the structure of the ventricular system, leading to the obstruction of key positions in the flow of CSF and impairing its normal movement. To unravel the causes of CH, it is essential to further explore the mechanisms governing the circulation of CSF. This represents a crucial foundation for advancing our understanding of this condition. It is noteworthy that not all cases of obstructive hydrocephalus can be attributed solely to abnormal ventricular structure development, infections, or tumors. Additional factors, such as abnormalities in glucose metabolism, altered expression of SCO-spondin protein, and disturbances in cell plane polarity, can also lead to impaired neurological development during embryonic stages, ultimately resulting in obstructive hydrocephalus. Investigating the underlying mechanisms behind these etiologies and exploring the potential development of medications and prenatal nutrients for pregnant women to safeguard against the development of CH in their fetuses would significantly contribute to hydrocephalus prevention efforts. This comprehensive approach holds promise for mitigating the incidence and impact of hydrocephalus.

## Data Availability

Not applicable.
